# Gastroduodenal Cryptococcus in an AIDS Patient Presenting With Melena

**DOI:** 10.4021/gr507w

**Published:** 2013-03-09

**Authors:** Yang Liu, Anish A. Patel, Janet C. Shaw, Eric P. Fillman, Paul B. Lamb

**Affiliations:** a Internal Medicine Department, Brooke Army Medical Center, San Antonio Uniformed Services Health Education Consortium (SAUSHEC), Ft. Sam Houston, TX, USA; b Gastroenterology Department, Brooke Army Medical Center, San Antonio Uniformed Services Health Education Consortium (SAUSHEC), Ft. Sam Houston, TX, USA; c Pathology Department, Brooke Army Medical Center, San Antonio Uniformed Services Health Education Consortium (SAUSHEC), Ft. Sam Houston, TX, USA

**Keywords:** *Cryptococccus neoformans*, Gastrointestinal Cryptococcosis, Stomach, Duodenum, Acquired Immunodeficiency Syndrome, HIV, Gastric ulcer

## Abstract

Gastrointestinal cryptococcosis is extremely rare with only a few case reports found in the literature and involvement primarily identified post-mortem. This is a case of 54-year-old man with a 20-year history of poorly controlled human immunodeficiency virus presented with constitutional symptoms along with melena. Diagnostic work up with esophagogastroduodenoscopy showed 4 irregular ulcers in the stomach notable for red-pigmented lesions within the ulcers, erythematous mucosa in the antrum and patchy friable mucosa in the duodenum. H&E staining and Mucicarmine staining showed findings consistent with *C. neoformans*. Blood culture and cerebrospinal fluid studies also revealed *C. neoformans*. *Cryptococcus neoformans* is an AIDS defining illness that most commonly presents as meningoencephalitis and pneumonitis. Key management principles includes: induction of antifungal therapy followed by consolidation and maintenance; management of elevated intracranial pressure and immune reconstitution inflammatory syndrome. Although the organism can infect nearly all organs, gastrointestinal involvement is rarely described. Our case highlights the fact that gastrointestinal *C. neoformans* infection can be associated with upper gastrointestinal symptoms and may be the initial presentation of disseminated cryptococcosis.

## Introduction

*Cryptococcus neoformans* (*C. neoformans*) is the most common systemic mycosis infection in patients with acquired immunodeficiency syndrome (AIDS) [1, 2]. The central nervous and pulmonary systems are the most frequent sites of infection [1, 2]. Gastrointestinal cryptococcosis is extremely rare with only a few case reports found in the literature and involvement primarily identified postmortem [3, 4]. We present a case of disseminated *C. neoformans* infection in an AIDS patient with gastrointestinal symptoms as the initial presentation and its associated endoscopic and pathologic findings.

## Case Report

A 54-year-old man with a 20-year history of poorly controlled human immunodeficiency virus (HIV), secondary to medication non-compliance, presented with an eight-day history of fever, malaise, and acute worsening of chronic watery diarrhea with melena. The patient had no history of NSAIDS use, denied headaches, mental status changes, and was without any neurological impairment. Vital signs were significant for heart rate of 112 beats per minute, blood pressure of 96/36 mmHg and fever to 100.4 Farenheit. Hemoglobin was 9.9 g/dL (14 - 18 g/dL), which was decreased by 3 g/dL from patient’s baseline; blood urea nitrogen and creatine was 48.6 mg/dL and 1.12 mg/dL respectively; and serum Helicobacter Pylori IGG was negative.

**Figure 1:**
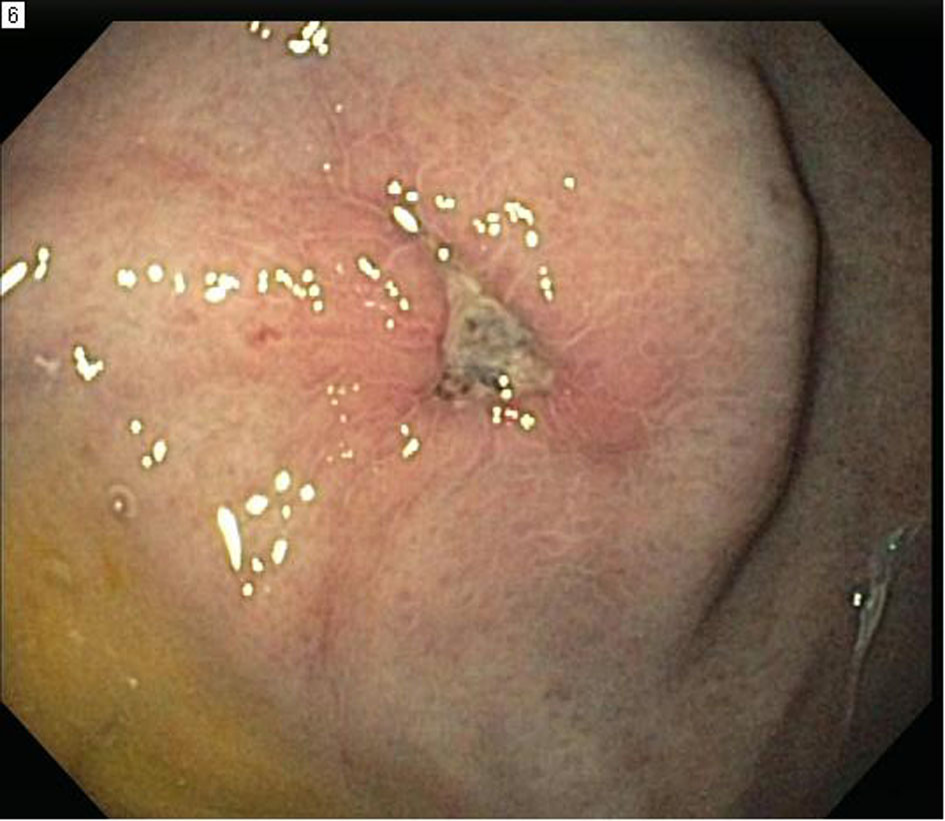
Endoscopic image showing large antral ulcer with central red pigmentation and surrounding erythema.

**Figure 2:**
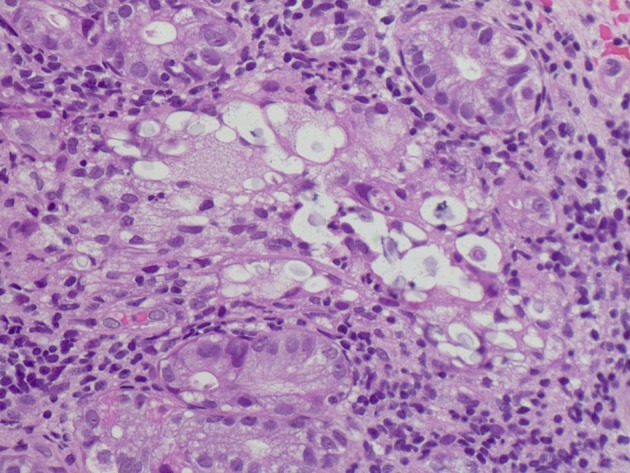
H&E staining: Extracellular varied sized round to oval organisms with a halo like clearing around each organism. 400 ×.

**Figure 3:**
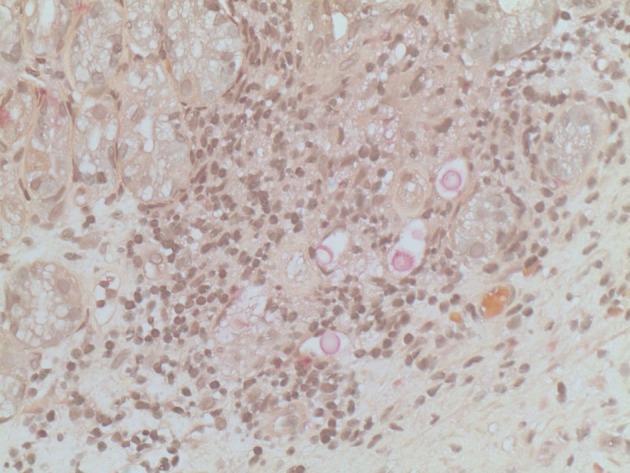
Mucicarmine staining: Pink staining highlights the organism’s mucopolysaccharide capsule, 400 ×.

**Figure 4:**
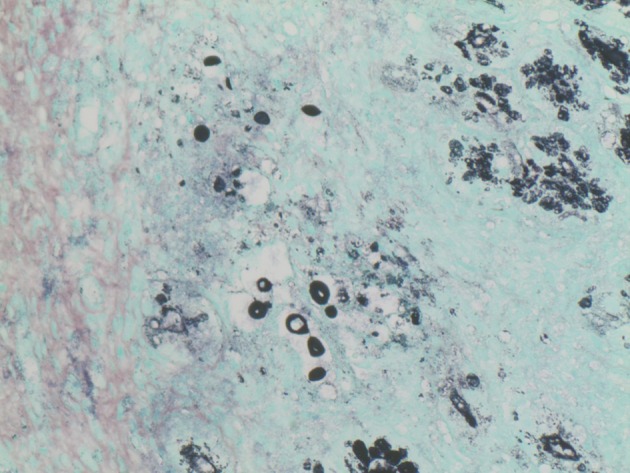
GMS staining: Black staining highlights the varied sized organisms and shows one organism with narrow based budding, 400 ×.

Esophagogastroduodenoscopy (EGD) was performed and showed 4 irregular ulcers in the stomach with the largest one notable for red-pigmented lesions within the ulcer (Fig. 1). Bipolar diathermy (bicap) was applied to 3 of the 4 ulcers and 3 endoclips were applied to the largest ulcer. The antral mucosa was moderately erythematous and the duodenal bulb and second part of the duodenum had patchy friable mucosa. Biopsies were obtained from the stomach and duodenum. H&E staining showed extracellular round to ovoid organisms, some of which had surrounding halo type clearing (Fig. 2). A mucicarmine stain highlighted the mucopolysaccharide capsule, consistent with *C. neoformans* (Fig. 3). A Grocott Methenamine Silver (GMS) stain decorated the fungal organisms and showed some narrow based budding (Fig. 4). Further testing revealed disseminated *C. neoformans* in the blood and cerebrospinal fluid. Patient was treated with amphotericin B followed by fluconazole as well as intravenous pantoprazole for treatment of *C. neoformans* and gastric ulcers. Unfortunately, he had a prolonged hospital course that was complicated by sepsis requiring broad spectrum antibiotics, intravenous fluid resuscitation and vasopressors. His clinical condition progressively deteriorated with worsening mental status and eventually passed away secondary to multisystem organ failure.

## Discussion

*Cryptococcus neoformans* is an AIDS defining illness, which typically causes disseminated disease in immunocompromised hosts and most commonly presents as meningoencephalitis and pneumonitis. CSF and serum antigen titer are also very specific and sensitive in the diagnosis of cryptococcal meningoencephalitis. Definitive diagnosis is made by culture of organism from cerebral spinal fluid (CSF). Other diagnostic findings included marked elevation of lumbar puncture opening pressure greater than 20 cm of CSF. Although there is no treatment specific for gastrointestinal cryptococcosis, the 2010 guidelines for the management of Cryptococcal disease by Infectious Disease Society of America recommend treating nonmeningeal, nonpulmonary cryptococcosis as central nervous system disease. Key management principles includes: 1). induction of antifungal therapy followed by consolidation and maintenance; 2). recognition and management of elevated intracranial pressure and immune reconstitution inflammatory syndrome (IRIS) [5]. Specific first line therapy for induction and consolidation include amphotericin B 0.7 - 1.0 mg/kg per day intravenously plus flucytosine 100 mg/kg per day orally in 4 divided doses; followed by fluconazole 400 mg (6 mg/kg) per day orally for a minimum of 8 weeks; and maintenance therapy with fluconazole 200 mg per day orally [5]. If the CSF pressure is greater than 25 cm of CSF and there are symptoms of increased intracranial pressure during induction therapy, reduce the opening pressure by 50% if it is extremely high or to a normal pressure of less than 20 cm of CSF. There is no need to alter direct antifungal therapy for minor IRIS manifestations and they will resolve spontaneously in days to weeks. However, consider corticosteroids for major manifestations, such as CNS inflammation with increased intracranial pressure [5].

*Cryptococcus neoformans* can infect nearly all organs, but gastrointestinal involvement is rarely described. In two series of 95 patients with AIDS and cryptococcosis, no gastrointestinal involvement was reported [1, 6]. Of the published reports of gastrointestinal cryptococcosis, the majority of the cases who were diagnosed postmortem by autopsy and were clinically silent, without documented endoscopic features [3, 4]. A thorough review of the literature revealed only 6 cases in which gastrointestinal symptoms were reported as the initial presentation in disseminated cryptococcosis. Two of the patients presented with acute abdomen requiring surgical intervention while the other reports presented with various symptoms to include odynophagia, diarrhea, nausea, and vomiting [3, 7-10]. In addition to the lack of endoscopic documentation, none of the reported cases of gastrointestinal cryptococcosis presented with upper gastrointestinal bleeding. We present a case of disseminated cryptococcosis in which melena was the primary clinical presentation and endoscopy aided in the diagnosis and management of the patient. Our case highlights that gastrointestinal *C. neoformans* infection can have associated endoscopic gastroduodenal mucosal changes. This report also emphasizes the fact that upper gastrointestinal symptoms, specifically melena, in AIDS patients may be the initial presentation of disseminated cryptococcosis and that endoscopy is a valuable tool in diagnosis and management.
